# Local ecological knowledge of the artisanal fishers on *Epinephelus itajara* (Lichtenstein, 1822) (Teleostei: Epinephelidae) on Ilhéus coast – Bahia State, Brazil

**DOI:** 10.1186/1746-4269-10-51

**Published:** 2014-06-25

**Authors:** Harildon M Ferreira, Gil M Reuss-Strenzel, Johnatas A Alves, Alexandre Schiavetti

**Affiliations:** 1 Programa de Pós-Graduação em Zoologia, Universidade Estadual de Santa Cruz, Rodovia Ilhéus/Itabuna km16, Ilhéus, Bahia, Brazil; 2 Departamento de Ciências Agrárias e Ambientais, Universidade Estadual de Santa Cruz – BA, Rodovia Ilhéus/Itabuna km16, Ilhéus, Bahia, Brazil; 3 Programa de Pós-Graduação em Sistemas Aquáticos Tropicais, Universidade Estadual de Santa Cruz, Rodovia Ilhéus/Itabuna km16, Ilhéus, Bahia, Brazil

**Keywords:** *Epinephelus itajara*, Local ecological knowledge, Reproductive aggregation, Endangered species, Marine protected areas

## Abstract

**Background:**

Local Ecological Knowledge (LEK) of traditional fishermen may be the only source of information regarding the conservation of the marine ecosystem and its endangered species. One of these species is *Epinephelus itajara*, which can exceed 2 m in length and 400 kg weight, is classified by the IUCN as a critically endangered. In Brazil, there is currently a moratorium that prohibits the capture of this specie, and in the northeastern coast, a Marine Protected Area was recently established properly justified by the existence a one spawning aggregation. The scope of the present study was the analysis the LEK of fishers with the goal of contributing to the conservation of *E. Itajara.*

**Methods:**

The Knowledge of 24 “experts” was recorded through semi-structured interviews with fishermen selected based on their expertise. LEK regarding some aspects of the life history of *E. itajara,* such as its morphology, spatial distribution, feeding, breeding and conservation, was systematized. The interviews were conducted in synchronic and diachronic situations. The data analysis followed the model of unity of the various individual skills, while the consistency of the analysis was tested using a matrix of methods employed in comparative cognitive science. Potential reproductive aggregation sites were identified by experts through projective interviews conducted based on a cartographic database and transferred to a geographic information system (GIS).

**Results:**

The LEK of these specialists in relation to the biological and ecological characteristics of *E. itajara* showed a high level of detail and a high agreement with the scientific literature. Projective interviews are presented as a promising tool allowing spatialization of the information generated through the registration of LEK. Therefore, the visualization of information from the fishermen, as well as its analysis and comparison with other databases, is simplified, thereby contributing to the decision-making process concerning the conservation of marine ecosystem in Brazil.

**Conclusions:**

Integration of LEK with scientific knowledge is an efficient strategy for the conservation of endangered species, as it provides important additional biological information that can be used in the process of participative and sustainable management of marine resources.

## Abstract (pt)

**Resumo (Portuguese):**

**Introdução**

O Conhecimento Ecológico Local (CEL) dos pescadores artesanais pode ser a única fonte de informações relativas à conservação do ecossistema marinho e suas espécies ameaçadas. Uma destas espécies é o *Epinephelus itajara*, podendo exceder 2 m de comprimento e 300 kg de biomassa, sendo que, é classificado pela UICN como Criticamente Ameaçada de Extinção. No Brasil existe atualmente uma moratória proibindo a captura desta espécie, e no Litoral Nordestino, uma Área Marinha Protegida foi recentemente decretada com a justificativa da existência de uma de suas agregações reprodutivas. O objetivo do presente estudo foi analisar o CEL dos pescadores para contribuir com a conservação do *E. itajara*.

**Metodologia**

O Conhecimento de 24 “especialistas” foi registrado através de entrevistas semi-estruturadas com pescadores selecionados por reconhecida experiência. O CEL foi sistematizado em relação a alguns aspectos da historia de vida do *E. itajara*, tais como: morfologia, distribuição espacial, alimentação, reprodução e conservação. Para verificar a validade das respostas recorreu-se a entrevistas repetidas em situações sincrônicas e diacrônicas. A análise dos dados seguiu o modelo de união das diversas competências individuais, e sua consistência foi testada com a construção de uma matriz de cognição comparada. Possíveis locais de agregação reprodutiva foram indicados pelos especialistas através de entrevistas projetivas realizadas sobre uma base cartográfica e transferida para um sistema de informações geográficas (sig).

O Conhecimento Ecológico Local destes especialistas em relação aos aspectos biológicos e ecológicos do *E. itajara* demonstrou alta correspondência e um nível refinado de detalhamento com a literatura cientifica especializada. As entrevistas projetivas se apresentam como ferramenta promissora, permitindo espacializar às informações geradas pelo registro do CEL. Assim, a visualização das informações provenientes dos pescadores, sua análise e comparação com outros tipos de base de dados são facilitadas, contribuindo para o processo de decisão relativo à conservação dos ecossistemas marinhos no Brasil.

**Conclusão**

A integração do CEL com o conhecimento científico pode ser uma eficiente estratégia de conservação para espécies ameaçadas, ao adicionar informações biológicas importantes para um processo participativo na gestão sustentável dos recursos marinhos.

## Background

Popularly known as “goliath grouper”, *Epinephelus itajara* is one of the largest Epinephelidae fish. This species can exceed 2 meters in total length and a weight of 400 kg during its lifetime expectancy of approximately 30 years [1-3]. The specie’s area of occurrence comprises coastal waters of the Atlantic Ocean, between Florida and southern Brazil (western shore) and between Senegal and the Congo (eastern shore) [4-6]. Some characteristics of this species’ life history and behavior increase its vulnerability to exploitation, especially spearfishing, such as: (i) slow growing with delayed maturity (can reach 37 years in age and maturing between 5 to 7 years, at 1 m total length) [7,8]; (ii) territorial fidelity (juvenile fishes live in mangroves and during adulthood is usually found on the continental shelf (<80 m) [9-15]; (iii) formation of spawning aggregations during the mating, (despite its solitary behavior, forming reproductive aggregations in specific areas and periods) [15,16]; (iv) show curiosity and fearlessness in approaching divers [17,18].

**Figure 1:**
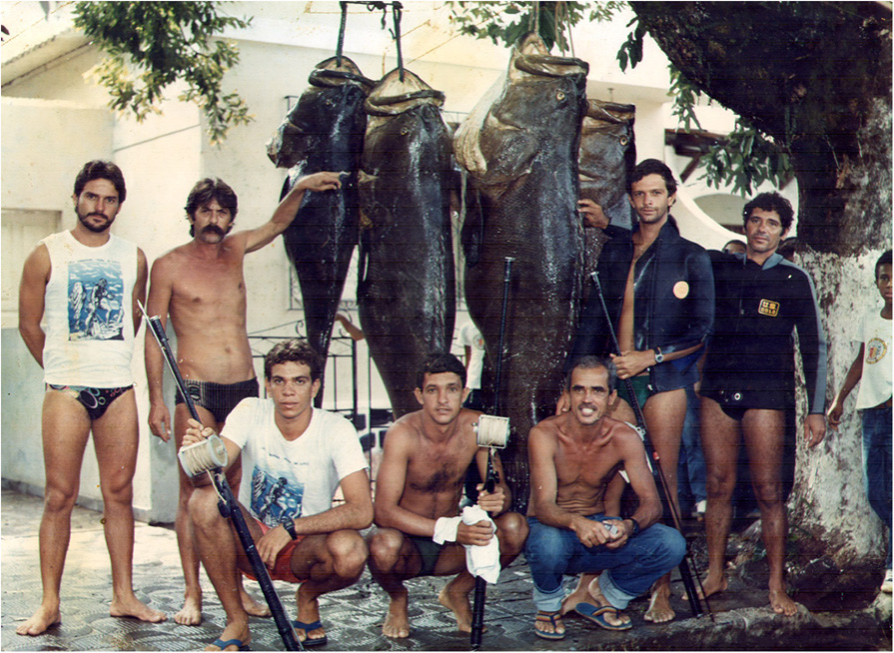
**Captures in reproductive aggregates.** Captures in Reproductive Aggregates in Ilhéus, in the 90′s (Photo: Demóstenes Bérbert Filho).

*E. itajara* is currently one of the most endangered fishes in the tropical Atlantic. The species is classified as critically threatened with extinction, as its population has experienced a rapid decline [19,20], old photos corroborate reports regarding the capture of large specimens in places where fisheries used to be abundant until the decade of the 1990′s (Figure 1). Fishing of goliath grouper has been banned in U.S. waters, the Gulf of Mexico and the Caribbean [2,16,21]. In Brazil, a moratorium that prohibits the capture of “mero” (Brazilian common name) until the year 2015 is currently in force; this period has been designated to allow the completion of research and projects that can support a national policy for the conservation of the species [22,23]. However, to be effective, this policy should consider supervision and management measures, such as the establishment of closed seasons and of Marine Protected Areas (MPA) [8,24,25].

In searching for a solution to the lack of technical information to preserve the marine ecosystem and its endangered species, scientists have been finding that the knowledge accumulated over generations by local fishermen can provide alternative useful source of data [26-30]. As fishermen must have knowledge of the variations in the environmental and biological cycles of fish resources, they often identify reproductive aggregations before scientists are able to [31-34]. The use of Local Ecological Knowledge (LEK) can assist in the planning of MPAs, as it provides information on the seasonality, quantity and size of individuals, as well as reproductive behavior and changes over time in the status of aggregates [35-38].

The few existing studies on *E. itajara* in Brazil have resulted from initiatives related to recording LEK based on reports from fishermen and divers, nevertheless, data indicate possible aggregation sites justify the creation of a MPA and supports another being currently considered [17,18,23,39]. The effective conservation of this species requires that more than a single aggregation is protected such that their reproductive success in ensured on study area. Therefore, for implementation of an MPA and the purpose of *E. itajara* conservation it’s necessary trustworthy information to an efficient decision-making process.

The goals of this study contribute for this process to analyze fishers’ LEK on *E. itajara* in respect to morphology, spatial distribution, feeding, breeding and conservation. In addition the fishermen also identified possible sites of spawning aggregations through projective interviews, this maps were transferred to a Geographic Information System (GIS) and serves as a tool to define strategies for the purpose of *E. itajara* conservation.

## Methods

### Study area

**Figure 2:**
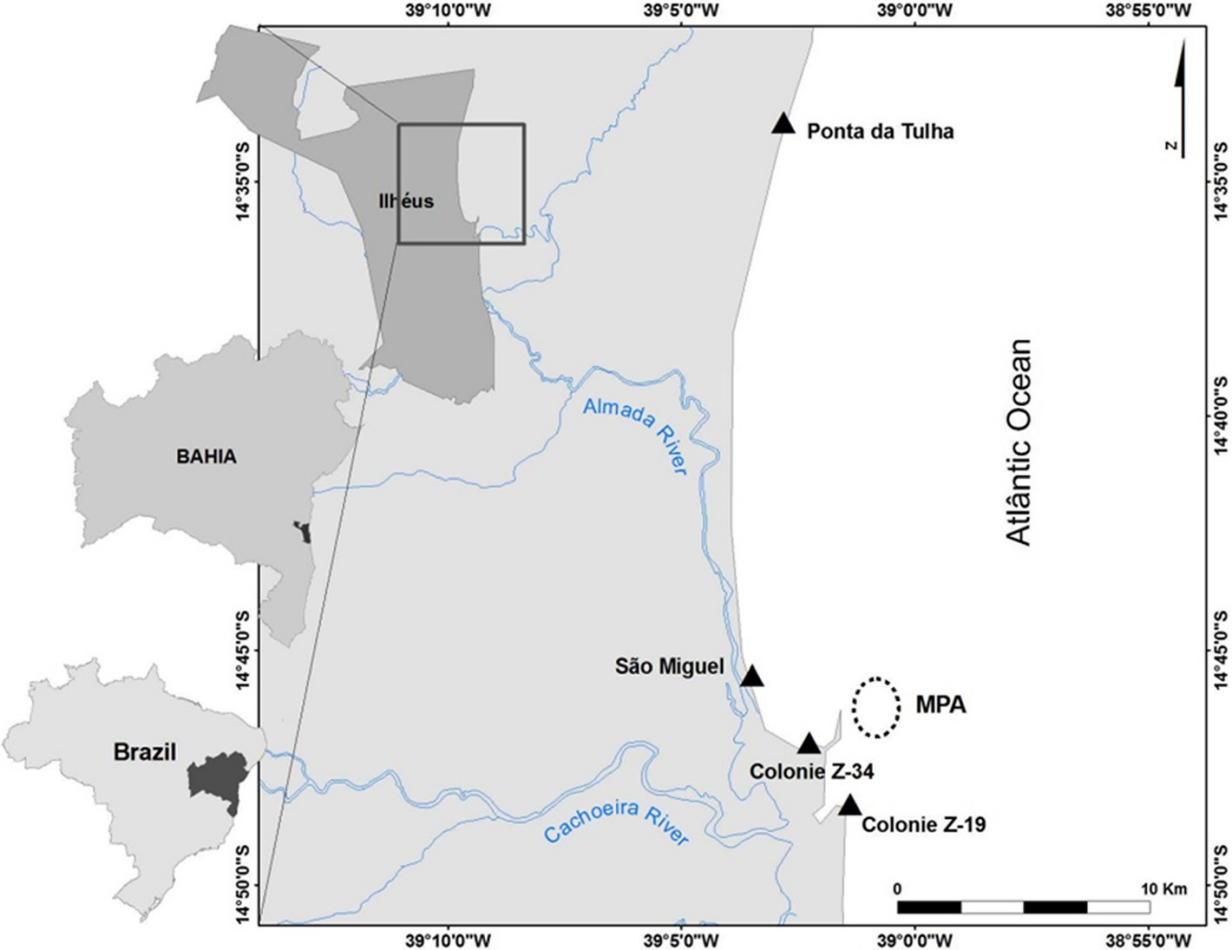
**Study area.** Location map of the city of Ilhéus - Bahia, with points selected for the conduction of interviews (triangles) and the area established by legal act as a Marine Protected Area (Circle).

The city of Ilhéus is located along the southern coast of Bahia, Northeastern Brazil (between meridians 39°00′ and 39°30′ W and parallels 14°20′ and 15° 00′ S) (Figure 2). The climate is characterized by a general pattern of atmospheric circulation related to the movement of the Divergence Zone of the South Atlantic high pressure cell. There are preponderant N and NE winds from October to March (summer) due to the Tropical Atlantic Mass and S and SE winds that occur most frequently from April to September (winter) according to the Atlantic Polar Front, as well as E trade winds that predominate periodically throughout the year [40-42].

The coastline of Ilhéus extends for approximately 80 km between the rivers Sargi to the north and Acuípe to the south [43]. The continental shelf in this region is relatively close to the 50 m and 200 m isobaths, which are located approximately 16 km and 17.5 km from the coastline, respectively. Sand and carbonatic mud is the predominant sediments close to various rocky outcrops and the continental shelf. Reef formations are present along the southern coast of Ilhéus [44].

The continental drainage system in the study area is mainly associated with the confluence of the Cachoeira, Santana and Fundão Rivers, which constitute the estuarine system of Pontal Bay. Other rivers also contribute to the process of coastal dynamics, such as the Almada River, which is located in the northern zone, and the Cururupe River, in the southern zone [45,46].

In front of Pontal Bay contains a large rocky reef that outcrops in some places, thereby forming “ilhéus” (islets on Portuguese), where the seabed extends from depths of 5 to 15 m. One *E. itajara* aggregation sites are located on this reef, which stimulated the creation of the MPA recently (Figure 2).

Currently in Ilhéus, there are two fishing colonies in the city: Z-19 and Z-34. The Z-19 colony was founded in 1921 and currently has 14 boats used for transportation in the colony. This colony includes approximately 1,500 members, 300 of which are active. The Z-34 colony was founded in 1947 and currently includes approximately 3,500 members, some of whom come from neighboring districts, including fishermen who work at sea, inland and in shellfish gathering in tidal waters.

The fishing conducted in study area is particularly artisanal due to the commercial production of small boats and with technological gap, which restricts activity on the continental shelf [47-49]. In relation to fishing gear, dominate the bottom line and logline used near to rocky substrate, for they are related to capture of large species with high commercial value such as groupers and snappers [49-51].

### Data collection and analysis

**Figure 3:**
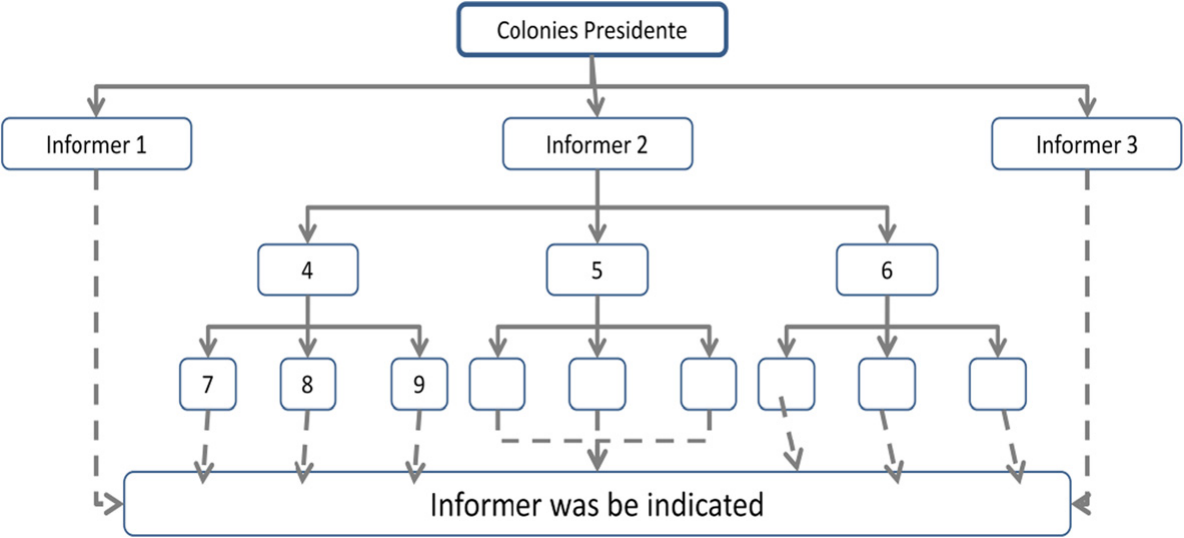
**Selection of experts.** Type of network information used for the selection of fishermen regarded as “experts” by members of the Z-19 and Z-14 fishing colonies.

Data were collected in the Z-19 and Z-34 colonies, whereas landing points were investigated in Sao Miguel and Ponta da Tulha neighborhoods, as the latter locations were used by local fishermen for trading activities and social gatherings and were close to their homes (Figure 2). Respondents were selected based on their expertise in overall fishing at this region [52-54], i.e., people who were renowned as culturally competent in their communities. Each of the presidents of the Z-19 and Z-34 fishing colonies indicated the three most experienced fishermen (informers), who indicated another three informers and so on. Fishermen who were indicated more than once (n ≥ 2) were therefore classified as experts and invited to participate in the study (Figure 3). Every expert was interviewed individually after they were introduced to the objectives of the project with the aid of a semi-structured questionnaire that was developed to facilitate systematization and analysis of data. All interviews were recorded with prior consent of the informants. This project is registered by the Ethics Committee, number 099/07, of the Estate University of Santa Cruz, Brazil.

To verify the consistency and validity of the responses, interviews were applied in synchronic and diachronic situations. The first type of interviews involve putting the same question to different people at times that are quite close to each other, whereas the second type involve repeating the same question to the same person at different times [55-58]. The data analysis followed the model of integrating different individual skills [55], during which all information obtained in the interviews was considered. Information on morphological characters, spatial distributions, feeding, breeding and conservation were compared. For the purpose of analyzing the consistency of local knowledge, comparative cognition tables were elaborated; the tables display information from fishermen and data from the scientific literature on the species side by side [58,59].

In addition to reporting some aspects of the life history of goliath grouper, the fishermen also identified possible sites of reproductive aggregations through projective interviews conducted on a cartographic basis [60]. The cartographic basis employed in the projective interviews was planned from the 1210 Nautical Chart [61]. Using this technique, information about the locations of known reproductive aggregation sites and places where captures have occurred was obtained. The resulting maps were transferred to a Geographic Information System (GIS) data base, where sites with a number of citations greater one (n ≥2) were considered as “potential sampling points” and classified according to the frequency of citations to be verified *in situ*.

To verify whether the number of interviews was satisfactory in relation to the locations referred to as hosting potential reproductive aggregations of *E. itajara*, a cumulative curve was produced. The data were randomized 500 times to abolish any influence of the interviews to avoid interfering with the pattern of the curve [62].

## Results and discussion

### Profile of expert fishermen

A total of 63 informants were registered, of which 33 were classified as experts, depending on the frequency of indications. Interviews were conducted with 24 experts, 11 of which came from Z-19 colony and 13 from Z-34 colony. Although at least three contacts were established with every selected fisherman for the purpose of building a reliable relationship, the reluctance of some of these individuals to provide information may be explained by the fear that the information would be used to justify regulatory or punitive measures. The situation could be aggravated by matters regarding endangered species legally protected, such as *E. itajara.*

The age of the interviewed fishermen ranged from 29 to 85 years, and their average age was 54 years. Over 70% of the interviewees learned to fish with their families, while the others learned from highly experienced older fishermen (Table 1). Information about the environment and its resources, as well as how to deal with them, is culturally transmitted [55]. The fishermen’s knowledge comes from their daily life and experiences shared from generation to generation [63].

**Table 1:** Profile of experts

**Time of fish experience**	**Number**	**Frequency (%)**
11-20 years	3	12.5
21-30 years	10	41.7
31-40 years	5	20.8
41-50 years	2	8.3
> 50 years	4	16.7
**Age class**		
< 40 years old	3	12,5
40-50 years old	8	33,3
51-60 years old	5	20,8
> 60 years old	8	33,3
**Initiations on fish**		
Family	17	70.8
Others	7	29.2
**Education level**		
Illiterate	3	12.5
Elementary school incomplete	18	75.0
Elementary school	2	8.3
High school	1	4.2
**Income source**		
Only fish	18	75.0
Fish and retirement	6	25.0

The education level of the study population was low: 75% had not concluded elementary school, and 12.5% were illiterate. These data are similar to findings from studies conducted both in the coastal region of Bahia [50,64,65] and other regions of Brazil [66], where most of the interviewed fishermen had an incomplete elementary education, and the others were illiterate or presented low education levels. According to several authors, the acceptance of local knowledge by scientists and managers is often limited by this socio-cultural barrier because they see this knowledge as a collection of superstitions and beliefs that are unreliable due to the lack of schooling of the people who hold them [59,67].

The source of income and financial support for 66.7% of the expert fishermen solely comes from the fish caught, while the others are retired due to old age, and fishing only complements their family income (Table 1). The respondents do not engage in other economic activities, as they are highly experienced and have skipper credentials. In contrast, several studies report that fishermen participate other economic activities in periods of low productivity, which are related to trade, civil construction, agriculture and service provision [50,68]. The different income sources recorded in the results presented here and in other reports can be again explained by the criterion adopted to select informants, as the most experienced fishermen are generally accredited as masters, which gives them economic advantages in the fishing industry. Some fishermen make their living solely from fishing, as they are acquainted with different fishing methods and strategies to collect other marine resources. In this study, all of the respondents were fishermen who use bottom lines as their main fishing gear; however, approximately 1/3 of them make use of other types of fishing gear at times when the “fish do not eat” (reduced capture), or when the oceanographic conditions are not favorable for angling (Table 2).

**Table 2:** Fishing characterization

**Time spent to a fishing trip**	**Number**	**Frequency (%)**
1 day	4	16.7
2 to 4 days	2	8.3
5 to 8 days	14	58.3
Fish no more	4	16.7
**Fishing gears**		
Only bottom-line	16	66.7
Bottom-line/gillnet	3	12.5
Bottom-line/gillnet/Log-line	3	12.5
Bottom-line/shrimp-trawling	2	8.3
**Seasonal preference**		
Winter	20	83.3
Summer	4	16.7

According to 100% of the interviewed fishermen, the seasonal variation in the region is defined solely in terms of summer and winter, and the difference is related to the direction and intensity of both wind and ocean currents. Despite the adversity reported in winter, when there are strong winds and high waves, it is the preferred time of the year by 83.3% of the experts with greater commercial value (Table 2).

“*Because the water stops running and we can catch more bottom fish*.”

Others authors [59] have analyzed traditional knowledge concerning aspects related to the temporal and spatial distribution of fishing resources and found that the fishermen also understand “winter” and the “summer” as the two main seasons affecting the abundance of fishing captures.

The time spent regarding the average duration of a fishing trip ranges from 5 to 8 days for most fishermen (58.3%), due to the short operating range of small motorboats, related to the capacity for storing ice, food and fuel. The boats of Ilhéus are still small and poorly equipped, hence hindering long-distance and deep water fishing, i.e., at depths greater than 200 meters [48,51].

### Analysis of local ecological knowledge

*E. Itajara* is popularly known as “*mero*” on the Brazilian coast [18,22], the fishermen of Ilhéus call this species “*mero-canapu*”; because the term “*mero*” is also used to classify other fishes of the Epinephelidae family. The designation “mero-canapu” was also reported by submarine hunters in the same city [17]. Only one expert reported a different nomenclature for the species, used when the fish presents yellowing or white coloring, which was “mero-flor-de-argodão” (*Cotton Flower Mero*). However, no other interviewee confirmed or reported knowledge of such a designation. Some also refer to this species as “*merete*” when it is a juvenile (small). According to Gerhardinger et al., [39], knowing the designation adopted by fishermen to refer to *E. itajara* is fundamental to the entire research process to ensure that both the researcher and the interviewee refer to the same species.

The constructed matrix of comparative cognition contains systematized information on the ecology of *Epinephelus itajara* with excerpts from interviews conducted with expert fishermen (Table 3). By comparing the information provided by experts with the available scientific literature, similarities can be noted between the two sources of knowledge.

**Table 3:** Matrix cognition compared

**Local ecological knowledge**	**Scientific literature**
**Morphological characteristics**	
“The mero-canapu have thicker scales, spurs, all thicker. It has the eye niggling, which is more darker brown then whiting and a little yellowish beneath the tip.”	Individuals of this species can be distinguished by morphological characters as small eye, wide mouth and coloration of the adult green with dark spots and bright [5,70].
“Big fish, the scales black with yellow and white flesh, round head”	
**Habitat**	
“He lives there, walks in the depth wall, is not a fish to walk so far on deep waters do not. He lives in the place that has a hole he is there, into those places of stone, is a bottom fish.”	Great copolymers are probably sedentary … they display little movement between reefs [14].
“On the stone edge, 50 m depth to around here. It’s fish that lives in rock, very dens, localized at the bottom near the dens.”	In his adulthood, is usually found in shallow waters of the continental shelf (<50 m) associated with various types of substrate, such as reefs, rock formations, shipwrecks and other similar structures [14,15].
“Experience in the rocks from outside and near the beach. In the stone which dens, is a bottom fish. The smaller (2–3 kg) we caught in the river back here (Almada River), 30 kg onwards is out there with 35 to 60 m deep.”	
“Find it in shallow and deep, but is created in the shallows”	The mangroves of the Florida, youth individuals gather in places with high structural complexity and unconsolidated funds [12].
**Diet**	
“ Eat all because it is a larger predator, eats octopus and various fish: tuna, small snappers and parrotfish, up when the animals already greater.”	The more important part of their diet is crustaceans, preferably lobster and crab, although fish and turtles were also found in their stomachs [2].
“The juvenil eats the same thing only smaller fish that takes”	Juveniles eat shrimp, crab and catfish [14].
“The small eat shrimp.”	
**Reproduction**	
“Come to shallow to spawn and then back to the depth”	
“The spawn is usually on the half moon …”	In Florida the goliath grouper breeding season is occurs from August to September, in time of full moon [7].
“In Itacare is spawning nest of them, once the boats took 5–6 a day. I even took three a day and three in another. In April and May we took there more than a hundred fish.”	In Belize and Puerto Rico have been recorded from July to August on the wreck to 30-45 m depth [2].
**Conservation**	
“ The mero came to Ilheus reef, in a place near the river where the young is so much more guarded and more food.”	The *E. itajara* is one of the most endangered fish in the tropical Atlantic [20].
“ Without spearfishing, it used to live longer, and it was just bigger, while today it reaches 70 or 80 kilograms and is shipped in large quantities.”	The main threats to *E. itajara* are anthropogenic, such as overfishing and habitat degradation [21].
“Who is beaten gets to learn, is not it! the fish going harpoon and receiving fish hook, is decreasing production. Many arts, include gillnet, also becomes more difficult to fishing.”	In Brazilian waters, the ban has been implemented for 5 years, during which time studies will address future management options [22].
“In handle bottom- line is rare, in shrimp trawl caught from time to time, and the diver kills everything.”	At aggregation sites off Florida numbers of fish again following the moratorium capture is increasing [2].
“You can be the net both to trawls and lobster, if started a few years back here. The fishnet destroys the stone from the bottom where small fish hide, and the great escape because it has little to eat.”	
“It’s not ending, and the population must have increased with the prohibition. That is the logic, isn’t it!”	

According to the interviewed fishermen, there is only a single species or variety of *E. itajara* on the coast of Ilhéus. This taxonomy can be considered as presenting a 1:1 correlation, i.e., when a single traditional generic taxon refers to a single scientific species [69]. The main criterion used by all 24 expert fishermen to describe mero was analysis of colour and body size (Table 4). Other citations refer to the head shape and the size of the mouth and scales, while only one mentioned the colour of meat (white) but related to an economic concern.

**Table 4:** Local knowledge on zoology

**Morphological characteristics**	**Number**	**Frequency (%)**
Body size	14	58.3
Staining pattern	24	100
Head shape	8	33.3
Size of the mouth	3	12.5
Scales	4	16.7
Sexual dimorphism	5	20,8
		
**Reproduction**		
Summer	13	54.2
Winter	4	16.7
Through the year	1	4.2
Did not respond	6	25
Simultaneously capture*	21	87.5

*captured more than a single individual in the same location and on the same day.

Most of the respondents (75%) did not identify sexual dimorphism in agreement with the available studies [70-72], and 20.8% could only identify the sex of the fish through visualization of the gonads (Table 4). Only one interviewee described morphological differences between males and females. Some species of fishin sex can be distinguished externally by color differences or by distinctive behaviors, although others species females may become so swollen with eggs [72].

“The female is always larger; from the umbilicus down it is paunchier than the male, and colour is the same.”

The maximum weight of Mero-canapu observed and/or captured by the expert fishermen when fishing of this species was allowed ranged from 48 to 300 kg (corresponding between 1.40 to 2.54 m in length) with an average of 147 kg (2.01 m). Captures were made in the marine area indicated by the fishermen as the favorite habitat of *E. itajara*. All of the fishermen described the presence of adult fishes in areas of rocky substrate on the continental shelf, where food and shelter were abundant (structural complexity); the following statements corroborate the findings presented in the scientific literature [1,14]:

“It lives in the rocks, but not everywhere, rocks near mud, on the bottom.”

“It’s a rocky fish, in the bottom close to the rocks, and it eats on the mud too.”

“A place with many stones, in the bottom where the fishes hide, it’s their home.”

According to 50% of the expert fishermen, adult and juvenile fishes are found in the same habitat (a); another, 29.2% of the respondents reported that juveniles have a preference for shallower sites (b), and that 20.8% of these stated that juveniles live in both the sea and in rivers (c), whereas only one reported that they are only found in rivers (Table 5). The remaining 12.5% did not respond.

**Table 5:** Local knowledge on environmental

**Habitat**	**Adult**	**Juvenile**
Marine	100	50
Estuary	4.2	25
Rock	100	66.7
Mud	8.3	20.8
Deep < 30 m	25	29.2
Deep > 30 m	100	50
**Diet**		
Fish	100	70.8
Lobster	37.5	25
Octopus	29.2	16.7
Shrimp	25	20.8
Did not respond	-	29.2
**Displacement**		
Foraging	58.3	-
Reproduction	41.7	-
Maturation	-	20.8
Predatory fishing	8.3	-

“Where the adult is, the cub is too.” (a)

*“I’ve caught little fishes in the same spot, but the big ones are found in deeper areas.”* (b)

*“Cubs are found into the river, and also at sea.”*(c)

The juveniles with total lengths reaching 1.0 m (approximately 18 kg) show a preference for the protected shallow water habitats associated with mangroves, which serve as shelter from predators and provide abundant food [11,12]. However, even the LEK which no associations were found in the literature can and must be complemented by scientific research for setting regulatory policies, such as the creation of the MPA in the Western Solomon Islands [73]. About the presence of juveniles on the continental shelf outside of mangroves, should not be discarded but rather be evaluated *in situ*, because the structural complexity of reefs can available shelter and food for some individuals in this stage lifecycle. Other research with LEK in southern Brazil, the informants were unable to differentiate locations where larger and smaller individuals are found [23].

With respect to the horizontal distribution of the species, displacement associated with different behaviors has been described to occur at the same depth and perpendicular to the shoreline. The most commonly referenced type of movement, cited by 58.3% of the respondents, was characterized as foraging (d), followed by behaviors associated with reproduction (e) and with maturation (f), cited by 41.7% and 20.8% of the fishermen, respectively (Table 5).

*“Every fish moves to find food. Yeah, it moves from one place to another, but it lives in the same place.”* (d)

*“He produces in the shallower rocks”* (e)

*“The small one dislocates through the continent for deep, while the big one spawns in shallow rocks near the beach.”* (e/f)

*”When it gets adult, it goes and makes its own life, but it is born in shallow areas.* (f)

According to Silvano & Begossi [30], fishermen from Buzios Island (Brazil) indicates that migration of *Epinephelus marginatus* (a closely related species of the genus) may be related to spawning aggregations, if fishermen did not mention such behavior, this remains an issue which deserves further investigation. It is suspected that the observed perpendicular displacement represents an ontogenetic migration in the life cycle of the species [15]. Two experts also described dislocations of Mero-canapu due to predatory fishing:

“Where have killed a fish the others runs away.”

“They keep on coming to the shallow areas, but they go to other rocks. Because have killed many fishes in the reef of Ilhéus.”

All of the respondents classified the *E. itajara* as an efficient and opportunistic predator and its main prey are fish, lobster and octopus (Table 5). The same food items were described for juvenile individuals of this species, with only variations in size of prey. Great similarities between the food items exploited by *E. itajara* in southern Brazil described from LEK [39] were the authors compared with the results with stomach contents of systematic ichthyological studies performed in the northern hemisphere [2], which also corroborates this study. In the absence of biological studies, Silvano & Begossi [30] does a good discussion about the potential of fishermen’s LEK to elaborate coastal food webs and their importance to understand ecological processes, such as cascade effects along the food chains which may be caused by the depletion of fishing stocks.

“It eats the big fishes; it is greedy and has a horrible mouth.”

“It loves eating lobster, octopus, and other fishes, such as the Yellowtail snapper and the horse-eyed jack.”

The influence of the moon and temperature on the eating habits of *E. itajara* was also described by the expert fishermen. According to the respondents, captures of *E. itajara* occur more often during the night and early in the day, depending on the temperature. During the new moon, the availability of live bait is reduced, as are the captures of most fishes caught using bottom lines, including Mero-canapu. Only two fishermen associated the full moon phase to the reproductive period of the species, thus corroborating the others studies performed in Brazil [17,39]. According to these previous studies, submarine hunters observe an increase in the abundance of fish associated with the full moon.

“When the sun is hot, fishes do not eat; they look for cooler waters.”

“When the moon is strong (full), fishes do not like to eat.”

“*Reproduction often occurs at the time of the half moon*.”

Concerning reproductive aspects, sometime in throughout the years of fishing activities the majority of fishermen (87.5%) witnessed a female *E. itajara* with mature gonads (Table 4). Most of the respondents said that it was during the summer, whereas five respondents provided different information: four said that reproduction occurs in winter, and one suggested that fish can reproduce throughout the year.

“I cannot say when, but that fish does not reproduce so much.”

“When fishes come from the cold deep waters, they just stay on the warm shallow waters for a little while; they spawn and leave.”

“There were many eggs in summer, the season of reproduction.”

Also there was no consensus among Búzios Island fishermen about the spawning season and the authors argue that disagreements may reveal new biological information suggesting that *E. marginatus* may have more than one spawning season along the Brazilian coast [30]. Despite the existence of isolated reports, it is internationally reported and accepted that *E. itajara* forms reproductive aggregations in summer, between June and October in the northern hemisphere [1,7,28] and from December to April in the southern hemisphere [17,18,23,39]. In Puerto Rico, goliath grouper post-larvae (15–18 cm) have been captured next to mangroves at the end of summer, indicating that summer is their reproductive period [10].

### Identification of reproductive aggregation areas

Seasonal aggregations are common in many fish species and occur for such reasons as feeding, migration and reproduction. Remembering that species has solitary behavior and territorial fidelity [1,2,15], the criteria for the identification of reproductive aggregation areas in this study are based on indirect evidences (number of individuals captured), i.e. obtained from the recording and analysis of information from experienced fishermen. This criterion is used here because it is not possible to recover information regarding the stage of maturity of the gonads, as the fish he himself caught are usually sold intact to the fish market, and the most of fisherman does not examine its viscera [39]. When asked about spawning aggregation sites, 87.5% of the experts confirmed that they had captured more than a single mero-canapu in the same location and on the same day (Table 4).

“We often see the couple, but we also see 3 or 4 together.”

“In the past, the boats caught about 5 or 6 in a single day. I myself have caught 3 in a single day and 3 in the next day.

In the absence of conclusive evidence, Gerhardinger et al. also consider the criterion of numbers of *E. itajara* observed in same area is a strong evidence of a spawning aggregation with LEK [23]. According to Colin et al., [72], to identify a reproductive aggregation with indirect signs there are three main criteria to be observed: a sudden increase in the number of individuals in a particular location, behavior and the physical characteristics of individuals indicate a spawn period, such as patterns of color and a distended abdomen.

**Figure 4:**
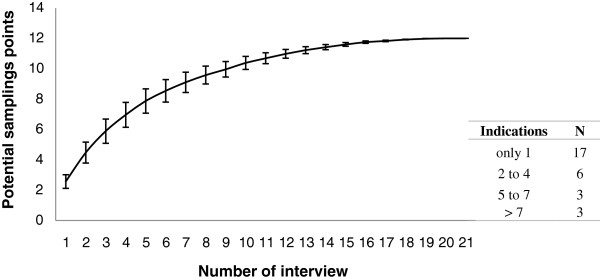
**Cumulative curve.** Cumulative curve for potential sampling points, mentioned by 21 of the “expert” fishermen during the projective interviews. The bars represent the lowest and highest values.

**Figure 5:**
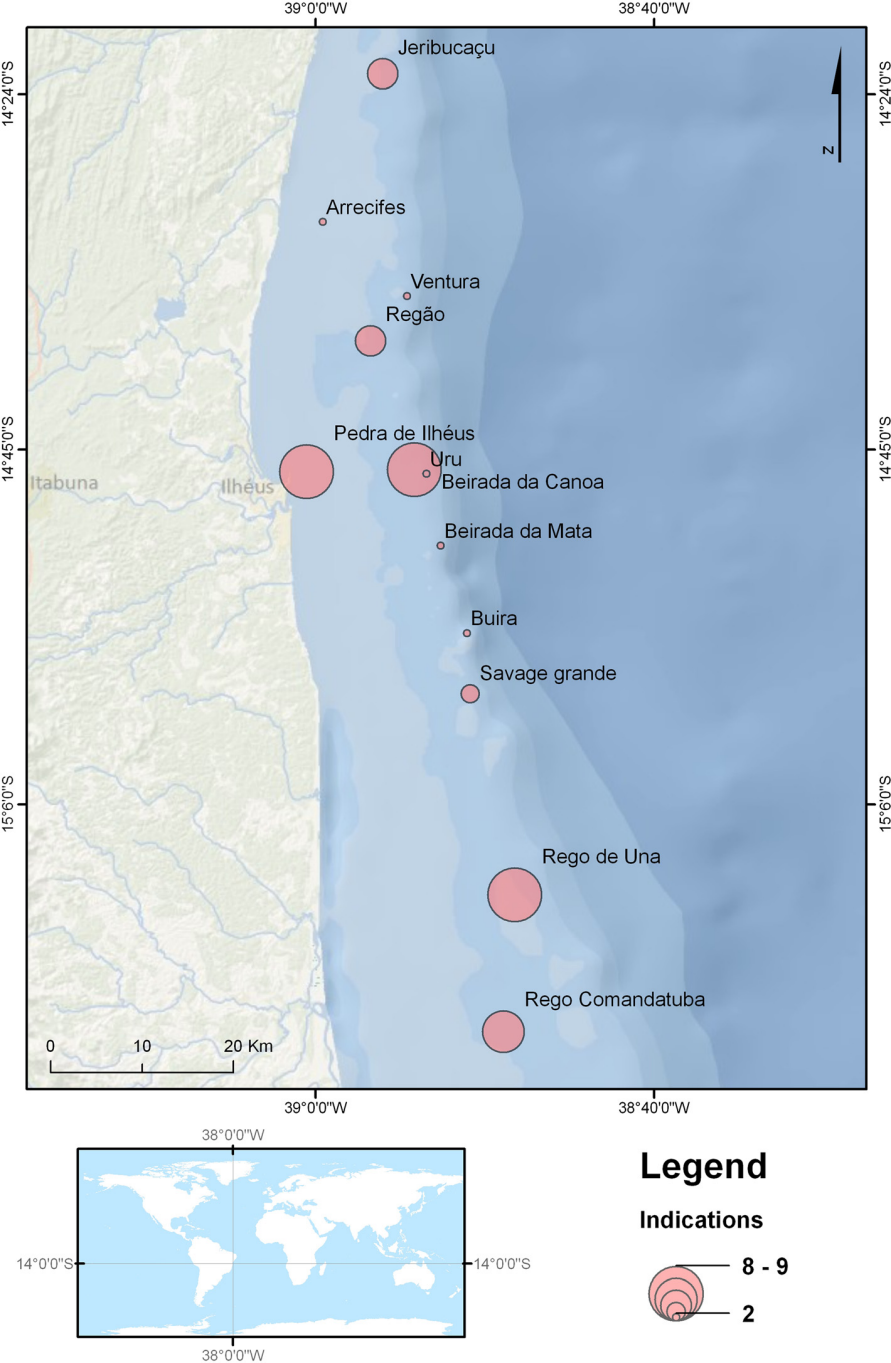
**Map indicated as potentials reproductive aggregations.** Map with areas indicated by expert fishermen as potentials reproductive aggregations of *E. itajara*.

Only three out of the twenty-four expert respondents could not perform the projective test. Two of the respondents fished on large ships along the northeastern coast and could not indicate the mero-canapu capture areas on the coast of Ilhéus, while the other could not identify the areas on the map. However, the number of projective interviews that have been conducted to date is satisfactory, as the number of identified potential sampling points began to stabilize from the sixteenth interview on the cumulative curve (Figure 4). The use of projective interviews allowed 29 areas where *E. itajara* was captured to be identified, 12 of which were cited by more than one specialist and, therefore, are classified according to the frequency of citations and therefore considered as sampling locations for later *in situ* verification. The vectorization of projective interviews resulted in the elaboration of a map showing the locations of capture points for the purpose of confirming or not if this is a reproductive aggregation sites (Figure 5).

The value of a good map of the aggregation site is obvious to conservation endangered species always aiming in implementation of marine resource management plans, such as coastal zoning and designing MPA [30,35,36,73,74]. Finding spawning aggregations in wide marine areas is rare; in addition, the costs of diving equipment and boat transport are significant. The most feasible option is therefore the use of the Local Ecological Knowledge (LEK) of fishers [35,75]. In Babitonga Bay, southern Brazil, Goliath grouper aggregations and relative abundances were described and mapped through the use of support the need for the MPA currently being considered [23]. In Ilhéus, northeastern Brazil, the creation of an MPA has been decreed justified by the existence of *E. itajara* aggregation sites, the records of which were obtained solely from the LEK of former submarine hunters [17]. Aswani & Lauer [76] also projective test used to identify the variation of the substrate and its associated fauna in the Western Solomon Islands. They considered correspondence between the surveys *in situ* and projective tests was good for many groups and added to the biological data should be used for MPA planning.

However, scientific verification is necessary to confirm the occurrence of these aggregations (Figure 5) as well as to study their ecological interactions and correlate them with any environmental factors. To confirm a reproductive event several direct signs can be recorded, such as females with eggs or hydrated post-ovulatory follicles or observation of the release of gametes in the water column [77,78]. Unverified reports of possible spawning aggregations are still valuable, but the limits of the data must always be part of the reporting [72].

### Local knowledge on conservation

The status of the population here was defined by the fishermen according to the temporal perception regarding the number of captured individuals. In most cases, the knowledge accumulated by local fishermen across generations is likely to be the only source of information about past conditions, considering the environmental changes and depletion of natural resources currently identified [26,29,31]. Among the experienced fishermen interviewed, 16.7% said that the capture of this species with bottom-line has always been rare, while 75% reported a large decrease in the number of *E. itajara* since they became involved with fishery (Table 6). Conversely, 8.3% believe that the current moratorium has allowed an increase in the population of mero-canapu to occur.

**Table 6:** Local knowledge on conservation

**Population status**	**Number**	**Frequency (%)**
Always been rare with Bottom-line	2	8.3
Decrease	15	62.5
Increase	4	16.7
Did not respond	3	12.5
**Impacts of fishing gear**		
Gillnet	7	29.2
Long-line	6	25.0
Spearfishing	12	50.0
Shrimp-trawling	3	12.5
Increase of outsiders ships	11	45.8
Conservation strategies*		
Inspection	15	62,5
Coastal zoning	5	20,8
Fish closed season**	3	12,5
Marine Protected Area**	7	29.2
No have solution	4	16,7

*recommendations from expert fishermen for the conservation of *E. itajara*.

**associate to other strategies.

“It is a hard to catch fish; its population is not decreasing; it only moves to different places.”

“It’s not ending, and the population must have increased with the prohibition. That is the logic, isn’t it!

“In the past, fishes were abundant. Three boats caught many fishes in a single spot. Today, no one can see that anymore.”

Some authors argue [33,79], when to fishes that resources are more abundant than biologists think, the first can claims of the former are self-serving but biologists have a responsibility not to dismiss such claims before investigating carefully. In Brazil, despite the current moratorium fishing effort is continuous, so in some regions the LEK registered indicate the decline both in the number of individuals and in the size of the captured fish, such us in northeastern over the decades has been reported [17,18] and in southern where the informants recalled a “good old days” [23,39].

When asked about the effects of fishing gear types on the integrity of the population of *E. itajara*, all of the fishermen admitted having made use of bottom lines and explained that such equipment could not have affected the species, as their capture rates were low. In the opinion of 33.3% of the interviewees, trawls provoke impacts, whereas 83.3% consider spearfishing as the main practice responsible for the large decline in the population of mero-canapu in the region (Table 6). Even the old submarine hunters, interviewed by Reuss-Strenzel & Assunção [17], consider owning up activity to represent the main responsible for the mero-canapu population decline, as this practice is still furtively. The same decline was described in Tamandaré [18], where the practice of spear fishing with scuba dive is an illegal activity but apparently quite common. According to 16.7% of the respondents, the population decline of this species is irreversible, as it is classified as threatened with extinction.

“It’s a fish we hardly catch… when using bottom-lines, the fish eats if it wants to eat.”

“When it leaves the reef to feed on the mud, it is caught by the trawl; I myself have caught a fish weighting 170 kg in that way.”

“Diving is a predatory fishing method; you catch the fish so forced in its own home.”

When the expert fishermen were asked to provide recommendations for the conservation of *E. itajara*, 62.5% of the expert respondents suggested that effective inspection by government agencies is necessary for fulfillment of the requirements of the legislation (Marinha do Brasil and IBAMA), especially the current moratorium and regarding the prohibition of spearfishing with the use of air compressors (Table 6). Additionally, 20.8% of the interviewees proposed the implementation of coastal zoning, under which each boat could only use marine resources in the federal state in which it was registered. Another regulatory measure proposed for 12.5% was the establishment of closed seasons for the protection of reproductive aggregations of mero-canapu during summer. Finally 29.2% support the establishment of marine protected areas that enable the reproduction and growth of both the *E. itajara* as the other species of commercial fish that are also decreasing.

“Boats from other regions must be prohibited, and fishes must have a free period for reproduction.”

The most appropriate conservation strategy for *E. itajara* is the creation of an MPA that protects its reproductive aggregations [2,36,80], as the species becomes more vulnerable in this period, and the fishing effort concentrates in the areas where these aggregations occur [17,77]. In Brazil, the existence of *E. itajara* aggregation sites justified a creation of an MPA that was been decreed [17] and another MPA currently being considered [23]. In other countries have additional examples of spawning sites that have gained protected-area status through the interaction of traditional fishers and researchers [73,76,80]. Again, MPAs emerge as an effective strategy that encompasses both the conservation of endangered species and the recovery of commercial fish stocks by protecting breeding and growth [75]. Lester et al. [81] analyzing 124 MPA found that positive responses (biomass, numerical density, species richness, and size of organisms inside MPA) are far more common than in differences or negative responses, validating the potential for well designed and enforced reserves to serve the globally important conservation and management tools. However, Schiavetti et al. [82] evaluated the Brazilian MPA found that there are few of these areas implemented in maritime range (up to 12 nautical miles) and the majority of existing areas do not have the protection of resources as the main goal.

Nevertheless indeed many researchers have acknowledged that they learned of the existence of spawning aggregations from fishers, and this is just one of many reasons why LEK involved from the start in the design and planning of marine reserves see [24,74,80]. A necessary step to democratic discussion regarding the importance and effectiveness of MPAs is, according Gerhardinger et al. [83], all stakeholders (scientists, government authorities, management councils and fishers) have a great deal of responsibility in integrating all the knowledge systems and thus have to follow ethical principle on the problem-solving.

Our intention with the results presented here is to contribute to the conservation of endangered species and also the fishery management on the coast of Bahia, considering both the fishers’ Local Ecological Knowledge and Marine Protected Areas.

## Conclusions

The methodology used for the selection of experts, in which every informant indicated three other fishermen, proved to be notably efficient. Through analyzing data regarding the profiles of the respondents, it can be inferred that the interviewees were actually experts in fishing and reliable informants because even though their education level was low, they were highly experienced in the use of marine resources as their main source of income. Additionally, the knowledge shared culturally by their ancestors or older experts has contributed to their proficiency.

The Local knowledge of these experts regarding the biological and ecological characteristics of *E. itajara* showed a refined level of detail and a high agreement with the scientific literature, thereby confirming the potential integration between these two knowledge systems - traditional ecological and scientific – that can be applied to the conservation of marine resources in Marine Protected Areas.

The cited information for which no associations were found in the literature should not be discarded but rather investigated to be reassessed and hence describe new behaviours and habits of this species.

Despite the fact that fishing *E. itajara* is prohibited, and its capture with bottom lines is considered rare, according to the LEK analysis, a considerable amount of information about this species is strongly present in the collective imagination, thus facilitating its use as a flagship species in the conservation strategy to protect marine biodiversity.

Projective interviews are seen as a promising tool, as they enable the spatialization of information generated through the registration of LEK. Thus, visualization of the information provided by fishermen and its analysis and comparison with other types of data are facilitated, thereby contributing to the decision-making process concerning the implementation of an MPA.

It is also necessary to confirm the existence of aggregations *in situ* to assess the conservation status of *E. itajara* and to validate projective interviews as a reliable tool for use in studies involving reproductive aggregates of fishes.

The point of Pedra de Ilhéus, where was created a MPA and identified on the projective interviews, is the only confirmed aggregations of *E. itajara*, consequently, it is necessary to conduct research in this area to obtain more knowledge concerning the ecology and reproductive behavior of this specie.

### Consent

Each fisherman was interviewed individually, after knowing the objectives of this report, with previous authorization of the informants through a "*Consent Term Free and Clarified*" to disclosure of information and pictures provided. Due to the level of schooling of fishermen, most terms were consented orally in recorded interviews and only a few were written. This research own registration with the Ethics Committee in the State University of Santa Cruz, under the number 099/07.

## Competing interests

The authors declare that they have no competing interests.

## Authors' contributions

HMF conceived the project, collected, organized and analyzed data and wrote the manuscript. GMRS coordinated the project, elaborated the map and contributed ideas and participated in discussions. AS contributed ideas to the study and discussion and was involved in revising the manuscript. JAA was involved in revising the manuscript. All authors read and approved the final manuscript.

## References

[B1] Bullock LH Murphy MD Godcharles MF Mitchell ME Age, growth, and reproduction of jewfish *Epinephelus itajara* in the eastern Gulf Mexico Fish Bull 1992 90 243 249

[B2] Sadovy Y Eklund AM Synopsis of biological data on the Nassau Grouper, Epinephelus striatus (Bloch, 1792), and the Jewfish, E. itajara (Lichtenstein, 1822) 1999 NOAA Technical report NMFS 146. FAO Fishery Synopsis 157

[B3] Morris AV Roberts CM Hawkins JP The threatened status of groupers (Epinephelinae) Biodivers Conserv 2000 9 919 942

[B4] Smith CL A revision of the Americam groupers: epinephelus and allied genera Bull Amer Mus Nat Hist 1971 146 69 241

[B5] Heemstra PC Randall JE FAO species catalogue: groupers of the world (Family Serranidae, Subfamily Epinephelinae) FAO Fish Synop 1993 16 125 382

[B6] Craig MT Graham RT Torres RA Hyde JR Freitas MO Ferreira BP Hostim-Silva M Gerhardinger LC Bertoncini AA Robertson DR How many species of goliath grouper are there? cryptic genetic divergence in a threatened marine fish and the resurrection of a geopolitical species Endanger Species Res 2008 13 1 8 preprint

[B7] Colin PL Preliminary investigations of reproductive activity of the jewfish, Epinephelus itajara (Pisces: Serranidae) Proceedings of the Gulf and Caribbean Fisheries Institute 1994 43 138 147

[B8] Coleman FC Koenig CC Huntsman GR Musick A Eklund AM Macgovern JC Chapman RW Sedberry GR Grimes CB Long-lived reef fishes: the grouper-snapper complex Fisheries 2000 25 3 14 20

[B9] Johnson GD Keener P Aid to identification of grouper larvae Bull Mar Sci 1984 34 106 134

[B10] Dennis GD Goulet D Roocker JR Hoyt RD Ichthyoplankton assemblages samples by night-light in nearshore habitat of southwestern Porto Rico Larval Fish Recruitment and Research in the Americas, Volume 95 1991 Mérida - México: NOAA Techn report NMFS

[B11] Laegdsgaard P Johnson C Why do juvenile fish utilize mangrove habitats? J Exp Mar Biol Ecol 2001 257 2 229 253 11245878 10.1016/s0022-0981(00)00331-2

[B12] Frias-Torres S Habitat use of juvenile goliath grouper Epinephelus itajara in the Florida Keys, USA Endanger Species Res 2006 1 1 6

[B13] Koenig CC Coleman FC Eklund AM Schull J Ueland J Mangroves as essential nursery habitat for goliath grouper (Epinephelus itajara) Bull Mar Sci 2007 80 3 567 586

[B14] Bullock LH Smith GB Seabasses (Pisces: Serranidae): memoirs of the hourglass cruises Florida Marine Research Institute 1991 VIII II

[B15] Eklund AM Schull J Sibert JR, Nielsen JL A stepwise approach to investigating the movement patterns and habitat utilization of Goliath Grouper, *Epinephelus itajara*, using conventional tagging, acoustic telemetry and satellite tracking Electronic Tagging and Tracking in Marine Fisheries 2001 The Netherlands: Kluwer Academic Publishers 189 215

[B16] Collins AB A preliminary assessment of the abundance and size distribution of goliath grouper *Epinephelus itajara* within a defined region of the central eastern Gulf of Mexico Proceedings of the Gulf and Caribbean Fisheries Institute 2009 61 184 190

[B17] Reuss-Strenzel GM Assunção MF Etnoconhecimento ecológico dos caçadores submarinos de Ilhéus, Bahia, como subsídio à preservação do mero (Epinephelus itajara Lichtenstein, 1822) Gerenciamento Costeiro Integrado 2008 8 203 219

[B18] Ferreira BP Maida M Projeto Mero: apresentação e resultados preliminares Boletim Técnico-Científico do CEPENE 1995 3 1 201 210

[B19] Lista Nacional das Espécies de Invertebrados Aquáticos e Peixes Ameaçadas de Extinção [http://www.mma.gov.br/estruturas/179/_arquivos/in_mma_005_04_179.pdf]

[B20] Red List of threatened animals [http://www.iucnredlist.org]

[B21] Service NMF Status Report on the Continental United States Distinct Population Segment of the Goliath Grouper (Epinephelus Itajara) 2006 USA: Florida

[B22] Hostim-Silva M Bertoncini AA Gerhardinger LC Machado LF The “Lord of the rock’s” conservation program in Brazil: the need for a new perception of marine fishes Coral Reefs 2005 24 1 74 74

[B23] Gerhardinger LC Hostim-Silva M Medeiros RP Matarezi J Bertoncini AA Freitas MO Ferreira BP Fishers’ resource mapping and goliath grouper *Epinephelus itajara* (Serranidae) conservation in Brazil Neotropical Ichthyology 2009 7 1 93 102

[B24] Lima JHM Dias-Neto J Ordenamento da pesca marítima no Brasil Boletim Técnico Científico – CEPENE, Tamandaré, Brasil 2002 10 1

[B25] Prates APL Cordeiro AZ Ferreira BP Maida M Unidades de Conservação marinha de uso sustentável como instrumento para a gestão pesqueira Áreas aquáticas protegidas como instrumento de gestão pesqueira 2007 Brasil: MMA, Brasília 25 38

[B26] Johannes RE The case for data-less marine resource management: examples from tropical nearshore finfisheries Trends Ecol Evol 1998 13 6 243 246 21238285 10.1016/s0169-5347(98)01384-6

[B27] Claro R Lindeman KC Spawning aggregation site of snapper and groupers species (Lutjanidae and serranidae) in the insular shelf of Cuba Gulf and Caribbean research 2003 14 2 91 106

[B28] Sala E Aburto-Oropeza O Paredes G Thompson G Spawning aggregations and reproductive behavior of reef fishes in the Gulf of California Bull Mar Sci 2003 72 1 103 121

[B29] Sáenz-Arroyo A Roberts CM Torre J Carino-Olvera M Using fishers’ anecdotes, naturalists’ observations and grey literature to reassess marine species at risk: the case of the Gulf grouper in the Gulf of California, Mexico Fish Fish 2005 6 6 121 133

[B30] Silvano RAM Begossi A Fishermen’s local ecological knowledge on Southeastern Brazilian coastal fishes: contributions to research, conservation, and management Neotropical Ichthyology 2012 10 133 147

[B31] Diegues AC Arruda RSV Saberes Tradicionais e Biodiversidade No Brasil 2001 Brasília, Brasil: Ministério do Meio Ambiente

[B32] Johannes B On the need for the study of indigenous fishers’ knowledge MPA news 2001 3 5 6

[B33] Johannes RE Freeman MMR Hamilton RJ Ignore fishers’ knowledge and miss the boat Fish Fish 2000 1 3 257 271

[B34] Begossi A Silvano RAM Ecology and ethnoecology of dusky grouper garoupa, epinephelus marginatus (Lowe, 1834) along the coast of Brazil J Ethnobiol Ethnomed 2008 4 20 18793394 10.1186/1746-4269-4-20 PMC2567293

[B35] Hamilton RJ Matawai M Potuku T Kama W Lahui P Warku J Smith AJ Applying local knowledge and science to the management of grouper aggregation sites in Melanesia SPC Live Reef Fish Information Bulletin 2005 14 7 9

[B36] Gerhardinger LC Freitas MO Medeiros RP Godoy EA Marenzi RC Hostim-Silva M Local Ecological Knowledge in the Planning and Management of Marine Protected Areas and in the Conservation of Fish Spawning Aggregations The Experience of Meros do Brasil Project Áreas Protegidas do Brasil 4 2007 Brasília, Brasil: MMA 107 129

[B37] Mutuku-Mathooko J Application of traditional ecological knowledge in the management and sustainability of fisheries in East Africa: a long-neglected strategy? Hydrobiologia 2005 537 1 6

[B38] Silvano RAM Begossi A Local knowledge on a cosmopolitan fish: ethnoecology of *Pomatomus saltatrix* (Pomatomidae) in Brazil and Australia Fish Res 2005 71 1 43 59

[B39] Gerhardinger LC Marenzi RC Bertoncini AA Medeiros RP Hostim-Silva M Local ecological knowledge on the Goliath grouper *Epinephelus itajara* (Teleostei: Serranidae) in Southern Brazil Neotropical Ichthyology 2006 4 4 441 450

[B40] Nimer E Climatologia do Brasil 1989 Rio de Janeiro, Brasil: IBGE

[B41] Dominguez JML Bittencourt ACSP Martin L Controls on quaternaly coastal evoluytion of the east-northeastern coast of Brazil: roles of sea-level history, trade winds and climate Sediment Geol 1992 80 213 232

[B42] Bittencourt ACSP Dominguez JML Martin L Silva IR Patterns of sediment dispersion coastwise the State of Bahia - Brazil An Acad Bras Ci 2000 72 2 271 287 10.1590/s0001-37652000000200012 10932122

[B43] França AMC Chaves HAF Geomorfologia da margem continental leste brasileira e das bacias oceânica adjacentes Reconhecimento global da margem continental brasileira (Projeto REMAC), Volume 7 1979 Rio de Janeiro, Brasil: Petrobrás/CENPES/DINTEP 89 128

[B44] Apoluceno DM A Influência do Porto de Ilhéus (BA) nos processos de acreção/erosão desenvolvidos após sua instalação 1998 Departamento de Geociências: Dissertação de Mestrado. Universidade Federal da Bahia

[B45] Superintendência de Recursos Hídricos do Estado da Bahia Diagnóstico das Bacias Hidrográficas dos Rios Cachoeira e Almada: Caracterização Climatológica. I (III) 2001 Salvador, BR: Relatório Técnico

[B46] Geremias R Características hidrográficas do sistema estuarino da Baia do Pontal 2002 Ilhéus: BA. Monografia de Especialização em Oceanografia Universidade Estadual de Santa Cruz. Ilhéus, BR

[B47] Centro de Pesquisa e Gestão de Recursos Pesqueiros do Litoral Nordeste Boletim Estatístico da Pesca Marítima e Estuarina do Nordeste do Brasil 2007 Tamandaré, PE: Monitoramento da atividade pesqueira no Estado da Bahia

[B48] Fernandes P Estudo da situação tecnológica da pesca artesanal Marítima de Peixes no Município de Ilhéus – Bahia. Dissertação de Mestrado 2003 Universidade Estadual de Santa Cruz. Programa de Pós-Graduação em Desenvolvimento Regional e Meio Ambiente

[B49] Lessa RP Oliveira JL Nóbrega MF Dinâmica das frotas pesqueiras da região Nordeste do Brasil. Programa de Avaliação do Potencial Sustentável dos Recursos Vivos da Zona Econômica Exclusiva (REVIZZE) 2004 Recife: Sub-Comitê Regional Nordeste (SCORE-NE). Relatório Síntese 131

[B50] Caló CFF Schiavetti A Cetra M Local ecological and taxonomic knowledge of snapper fish (Teleostei: actinopterygii) held by fishermen in Ilhéus, Bahia, Brazil Neotropical Ichthyology 2009 7 3 403 414

[B51] Barbosa-Filho MLV Cetra M Dinâmica da frota pesqueira sediada na cidade de Ilhéus, Estado da Bahia Boletin Técnico Cientifico 2007 15 2 99 105

[B52] Marques JGW Pescando Pescadores: Etnoecologia Abrangente no Baixo São Francisco 1995 São Paulo: NUPAUB-USP

[B53] Davis A Wagner JR Who knows? on the importance of identifying “experts” when researching local ecological knowledge Hum Ecol 2003 31 3 463 489

[B54] Alarcon DT Schiavetti A O Conhecimento dos Pescadores Artesanais de Itacaré (BA) sobre a Fauna de Vertebrados (não peixes) Associados às Atividades Pesqueiras Revista Gerenciamento Costeiro Integrado 2005 4 1 4

[B55] Marques JGW Aspectos Ecológicos na Etnoictiologia dos Pescadores do Complexo Estuarino-lagunar Mundaú-Manguaba 1991 UNICAMP: Tese de Doutorado

[B56] Costa-Neto EM Marques JGW Atividades de pesca desenvolvidas por pescadores da comunidade de Siribinha, município de Conde, Bahia: uma abordagem etnoecológica Sitientibus Série Ciências Biológicas 2001 1 1 71 78

[B57] Mourão JS Nordi N Pescadores, peixes, espaço e tempo: uma abordagem etnoecológica Interciencia 2006 31 5 1 7

[B58] Silvano RAM Begossi A Pesca artesanal e etnoictiologia Ecologia de Pescadores da Mata atlântica e da Amazônia 2004 São Paulo: Hucitec 185 220

[B59] Costa-Neto EM Marques JGW Conhecimento ictiológico tradicional e a distribuição temporal e espacial de recursos pesqueiros pelos pescadores de Conde, Estado da Bahia, Brasil Etnoecológica 2000 4 6 56 68

[B60] Minayo MCS Pesquisa Social: Teoria, Método e Criatividade 1996 6ª Petrópolis: Vozes

[B61] Diretoria de Hidrografia e Navegação Carta Náutica 1201: Porto do Malhado - Ilhéus. Escala 1:12.500 1989 Marinha do Brasil In press

[B62] Colwell RK Estimate S Statistical estimation of species richness and shared species from samples: version 7 2005 User’s Guide and application

[B63] Paz VA Begossi A Ethnoichthyology of Gamboa: fishermen of Sepetiba bay, Brazil J Ethnobiol 1996 16 2 157 168

[B64] Pacheco RS Aspectos da ecologia de pescadores residentes na península de Maraú, Bahia: pesca, uso de recursos marinhos e dieta 2006 Dissertação de Mestrado: Universidade de Brasília, Departamento de Ecologia

[B65] Damaso RCSC Etnoecologia dos pescadores de Itacaré, BA 2006 Dissertação de Mestrado. Universidade Estadual de Santa Cruz. Programa de Pós-Graduação em Desenvolvimento Regional e Meio Ambiente

[B66] Garcez DS Sánchez-Botero JI Comunidades de pescadores artesanais no estado do Rio Grande do Sul, Brasil Revista Atlântica 2005 27 1 17 29 In press.

[B67] Baelde P Using Fishers’ Knowledge Goes Beyond Filling Gaps in Scientific knowledge - Analysis of Australian Experiences Proceedings Putting Fishers’ Knowledge to Work Conference 2001 Vancouver, Canada: University of British Columbia 78 86

[B68] Alarcon DT Costa RCS Schiavetti A Abordagem Etnoecológica da pesca e captura de espécies não-alvo em Itacaré, Bahia (Brasil) Boletim do Instituto de Pesca CEPENE 2009 35 663 674

[B69] Figueiredo JL Menezes NA Manual de peixes marinhos do sudeste do Brasil. III Teleostei (2) 1980 São Paulo, Brazil: Museu de Zoologia da Universidade de São Paulo

[B70] Berlin B Folk systematics in relation to biological classification and nomenclature Annu Rev Ecol Syst 1973 4 259 271

[B71] Heemstra PC Randall E FAO species catalogue: groupers of the world (Family serranidae, subfamily epinephelinae) FAO Fish Synop 1993 16 125 382

[B72] Colin PL Sadovy YJ Domeier ML Manual for the study and conservation of reef fish spawning aggregations Society for the Conservation of Reef Fish Aggregations Special Publication 2003 1 1 98

[B73] Aswani S Hamilton RJ Integrating indigenous ecological knowledge and customary sea tenure with marine and social science for conservation of bumphead parrotfish (Bolpometodon muricatum) in the Roviana Lagoon, Solomon Islands Environ Conserv 2004 31 1 69 83

[B74] Gell FR Roberts CM Benefits beyond boundaries: the fishery effects of marine reserves TRENDS in Ecology and Evolution 2003 18 9 448 455

[B75] Drew JA Use of traditional ecological knowledge in marine conservation Conserv Biol 2005 19 4 1286 1293

[B76] Aswani S Lauer M Benthic mapping using local aerial photo interpretation and resident taxa inventories for designing marine protected areas Environ Conserv 2006 33 263 273

[B77] Koenig CC Coleman FC Grimes CB Fitzhugh GR Scalon KM Gledhill CT Grace M Protection of fish spawning habitat for the conservation of warm-temperate reef-fish fisheries of sheledge reefs of Florida Bull Mar Sci 2000 66 593 616

[B78] Domeier M Colin PL Tropical reef fish spawning aggregation defined and reviewed Bull Mar Sci 1997 60 3 698 726

[B79] Silvano RAM Valbo-Jørgensen J Beyond fishermen’s tales: contributions of fishers’ local ecological knowledge to fish ecology and fisheries management Environ Dev Sustain 2008 10 657 675

[B80] Johannes RE Squire L Granam T Sadovy Y Renguul H Spawning Aggregations of Groupers (Serranidae) in Palau 1999 Marine Conservation Research Series Publ.#1, The Nature Conservancy 144

[B81] Lester SE Halpern BS Grorud-Colvert K Lubchenco J Ruttenberg BI Gaines SD Airamé S Warner RR Biological effects within no-take marine reserves:a global synthesis Mar Ecol Prog Ser 2009 384 33 46

[B82] Schiavetti A Manz J Santos CZ Magro TC Pagani MI Marine protected areas in Brazil: an ecological approach regarding the large marine ecosystems Ocean & Coastal Management 2013 76 96 104

[B83] Gerhardinger LC Godoy EAS Jones PJS Local ecological knowledge and the management of marine protected areas in Brazil Ocean & Coastal Management 2009 52 154 165

